# 4-{5-[(2-Bromo­benz­yl)sulfan­yl]-1*H*-tetra­zol-1-yl}benzoic acid

**DOI:** 10.1107/S1600536813014840

**Published:** 2013-06-12

**Authors:** Ana C. Mafud, Yvonne P. Mascarenhas, Alessandro S. Nascimento

**Affiliations:** a Instituto de Física de São Carlos, Av. do Trab. Sãocarlense, 400, São Carlos, SP, Brazil

## Abstract

In the title compound, C_15_H_11_BrN_4_O_2_S, the tetra­zole ring makes dihedral angles of 45.97 (10) and 75.41 (1)°, respectively, with the benzoyl and bromo­benzene rings while the dihedral angle between the benzene rings is 73.77 (1)°. In the crystal, mol­ecules are linked through O—H⋯ N and C—H⋯ O hydrogen bonds, giving infinite chains in both the [110] and [1-10] directions. These chains are further connected by C—Br⋯π and C—O⋯π inter­actions and also by π–π stacking between tetra­zole rings [centroid–centroid distance = 3.312 (1) Å], generating a three-dimensional network.

## Related literature

For details of the ZINC database, see: Irwin *et al.* (2012 ▶). For biological properties of tetra­zoles, see: Kees *et al.* (1989 ▶); Nolte *et al.* (1998 ▶); Mafud *et al.* (2013 ▶).
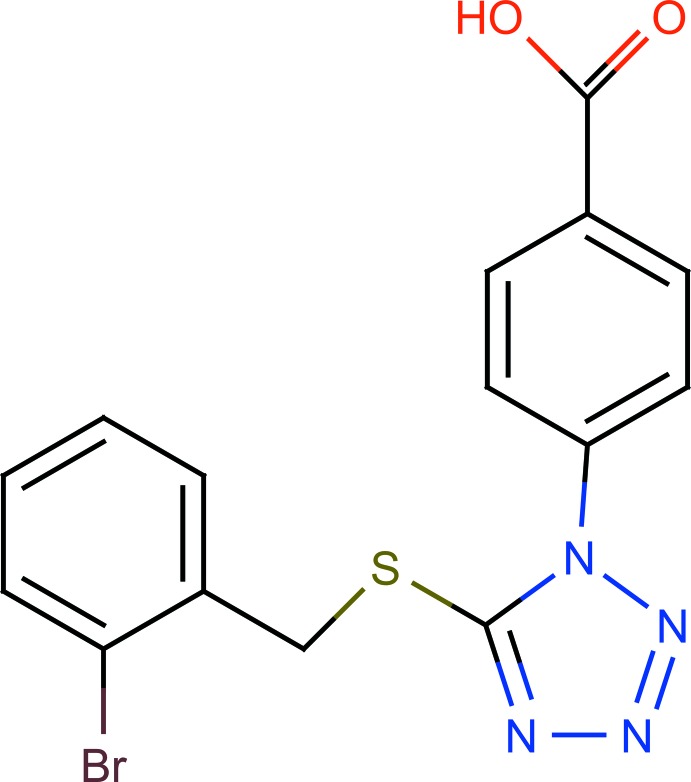


## Experimental

### 

#### Crystal data

C_15_H_11_BrN_4_O_2_S
*M* *_r_* = 391.25
Monoclinic, 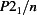

*a* = 7.4570 (5) Å
*b* = 8.3500 (5) Å
*c* = 25.3680 (14) Å
β = 97.626 (3)°
*V* = 1565.59 (17) Å^3^
*Z* = 4
Mo *K*α radiation
μ = 2.77 mm^−1^
*T* = 290 K
0.1 x 0.1 (radius) mm

#### Data collection

Nonius KappaCCD diffractometer
Absorption correction: for a cylinder mounted on the ϕ axis (modified Dwiggins, 1975 ▶) *T*_min_ = 0.604, *T*_max_ = 0.608
24464 measured reflections
2889 independent reflections
2388 reflections with *I* > 2σ(*I*)
*R*_int_ = 0.069

#### Refinement

*R*[*F* ^2^ > 2σ(*F* ^2^)] = 0.041
*wR*(*F* ^2^) = 0.114
*S* = 1.06
2889 reflections
213 parameters
H atoms treated by a mixture of independent and constrained refinement
Δρ_max_ = 0.38 e Å^−3^
Δρ_min_ = −0.47 e Å^−3^

### 

Data collection: *COLLECT* (Nonius, 1999 ▶); cell refinement: *SCALEPACK* (Otwinowski & Minor, 1997 ▶); data reduction: *DENZO* (Otwinowski & Minor, 1997 ▶) and *SCALEPACK*; program(s) used to solve structure: *SIR92* (Altomare *et al.*, 1994 ▶); program(s) used to refine structure: *SHELXL97* (Sheldrick, 2008 ▶); molecular graphics: *ORTEP-3 for Windows* (Farrugia, 2012 ▶) and *Mercury* (Macrae *et al.*, 2008 ▶); software used to prepare material for publication: *WinGX* (Farrugia, 2012 ▶).

## Supplementary Material

Crystal structure: contains datablock(s) I, global. DOI: 10.1107/S1600536813014840/lr2105sup1.cif

Structure factors: contains datablock(s) I. DOI: 10.1107/S1600536813014840/lr2105Isup2.hkl

Supplementary material file. DOI: 10.1107/S1600536813014840/lr2105Isup3.cml

Additional supplementary materials:  crystallographic information; 3D view; checkCIF report

## Figures and Tables

**Table 1:** Hydrogen-bond geometry (Å, °) *Cg*1 and *Cg*2 are the centroids of the N1–N4/C8 tetra­zole ring and the C10–C15 benzene ring, respectively.

*D*—H⋯*A*	*D*—H	H⋯*A*	*D*⋯*A*	*D*—H⋯*A*
C9—H9*B*⋯O1^i^	0.97	2.41	3.351 (4)	163
O2—H1⋯N4^ii^	0.73 (4)	2.05 (4)	2.746 (3)	161 (5)
C1—O1⋯*Cg*1^iii^	1.21 (1)	3.62 (1)	4.534 (1)	133 (2)
C11—Br1⋯*Cg*2^iv^	1.90 (1)	3.58 (1)	4.895 (2)	124 (1)

Symmetry codes: (i) 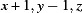; (ii) 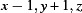; (iii) 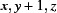; (iv) 
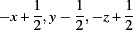
.

**Fig. 1.**
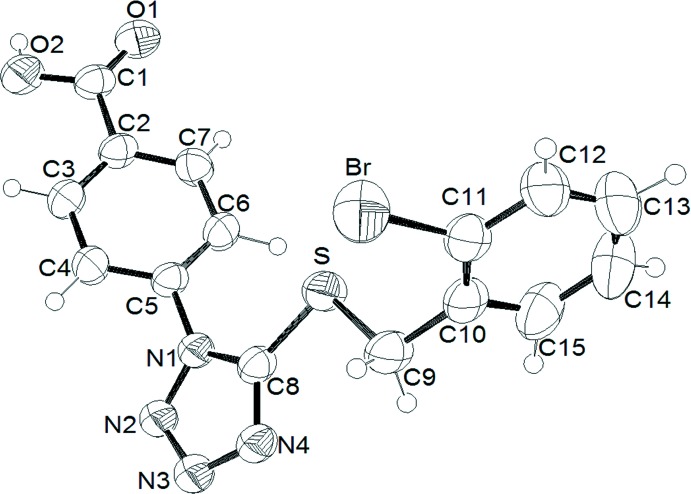
Perspective view of the molecular structure of the title compound, with displacement ellipsoids drawn at the 50% probability level.

**Fig. 2.**
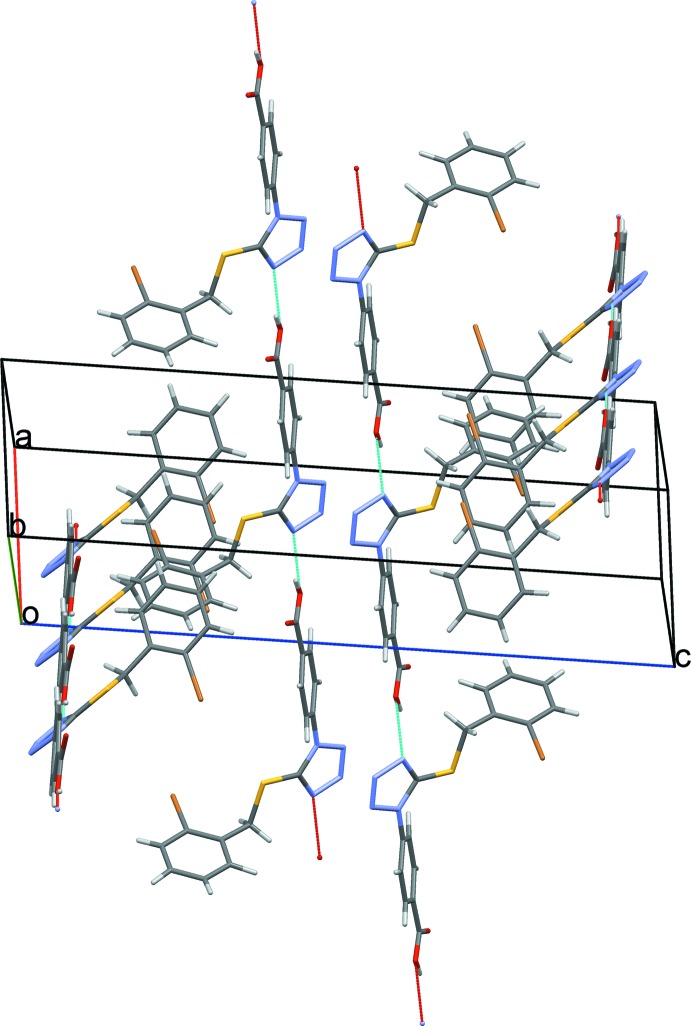
A view along the a axis of the crystal packing of the title compound.

## supplementary crystallographic information

### Comment

The title acid is a screening molecule available in the ZINC database (Irwin *et al.*, 2012) among the 'drugs-now' subset. This molecule has been identified as a PPAR gamma ligand candidate in a virtual screening study. The Peroxisome Proliferator-Activated Receptor, isoform gamma, is a transcription factor that regulates the expression of genes involved in glucose and lipid metabolism (Nolte *et al.*, 1998). Our group recently described the crystal structure of a similar compound, evaluated as a PPARgligand in a competition assay (Mafud *et al.*, 2013). Since tetrazoles are already known to have glucose lowering effects *in vivo* (Kees *et al.*, 1989), in this virtual screening we chose some different representative molecules to evaluate the affinities and the extent of receptor activation. Here, we report the crystal structure of the title compound.

The dihedral angles between the tetrazole and the benzoyl and the bromo benzene rings are 45.97 (10)° and 75.41 (1)° respectively, while between the two benzene rings is 73.77 (1)°. The crystal packing is established through interactions of three types (Table 1):

The first one are intermolecular hydrogen bonds: O2—H1···N4(ii) and C9—H9···O1(i), with symmetry codes: (i)1+ *x*, -1 + *y*, *z* and (ii) -1+*x*, 1+*y*, *z*

The second one are C–*X*···π interactions: C1—O1 ··· *Cg*1(iii), 3.619 (2) Å and C11—Br1 ··· *Cg2*(iv), 3.581 (2) Å, where *Cg*1 and *Cg*2 are the centroids of the {N1, N2, N3, N4, C8} and {C10, C11, C12, C13, C14, C15} rings, respectively, with symmetry codes: (iii) *x*, 1 + *y*, *z*; (iv) 1/2 - *x*,-1/2 + *y*,1/2 - *z*.

And the last type is π-π stacking between centrosymmetric tetrazole neighbour rings (centroid-centroid distance=3.312 (1) Å )

### Experimental

A colourless single-crystal of the title compound was selected from the sample as supplied (Pharmeks Ltd.) without recrystallization.

### Refinement

The hydroxyl H atom was located in a difference Fourier map and refined with *U*_iso_(H) = 1.5Ueq(O). The C-bound H atoms were included in calculated positions and treated as riding atoms: C—H = 0.93 and 0.97 Å, for CH and CH_2_ respectively, with *U*_iso_(H) = 1.2Ueq(C).

### Crystal data

**Table:**

C_15_H_11_BrN_4_O_2_S	*F*(000) = 784
*M**_r_* = 391.25	*D*_x_ = 1.66 Mg m^−^^3^
Monoclinic, *P*2_1_/*n*	Mo *K*α radiation, λ = 0.71073 Å
Hall symbol: -P 2yn	Cell parameters from 3980 reflections
*a* = 7.4570 (5) Å	θ = 12.0–18.1°
*b* = 8.3500 (5) Å	µ = 2.77 mm^−^^1^
*c* = 25.3680 (14) Å	*T* = 290 K
β = 97.626 (3)°	Prism, colourless
*V* = 1565.59 (17) Å^3^	0.1 × 0.1 × 0.1 × 0.1 (radius) mm
*Z* = 4	

### Data collection

**Table:**

Nonius KappaCCD diffractometer	2889 independent reflections
Graphite monochromator	2388 reflections with *I* > 2σ(*I*)
Detector resolution: 9 pixels mm^-1^	*R*_int_ = 0.069
CCD scans	θ_max_ = 25.7°, θ_min_ = 2.9°
Absorption correction: for a cylinder mounted on the φ axis (Dwiggins, 1975)	*h* = −9→9
*T*_min_ = 0.604, *T*_max_ = 0.608	*k* = −10→10
24464 measured reflections	*l* = −30→30

### Refinement

**Table:**

Refinement on *F*^2^	Primary atom site location: structure-invariant direct methods
Least-squares matrix: full	Secondary atom site location: difference Fourier map
*R*[*F*^2^ > 2σ(*F*^2^)] = 0.041	Hydrogen site location: inferred from neighbouring sites
*wR*(*F*^2^) = 0.114	H atoms treated by a mixture of independent and constrained refinement
*S* = 1.06	*w* = 1/[σ^2^(*F*_o_^2^) + (0.0623*P*)^2^ + 0.5855*P*] where *P* = (*F*_o_^2^ + 2*F*_c_^2^)/3
2889 reflections	(Δ/σ)_max_ < 0.001
213 parameters	Δρ_max_ = 0.38 e Å^−^^3^
0 restraints	Δρ_min_ = −0.47 e Å^−^^3^

### Special details

**Table:**

Experimental. Absorption correction: interpolation using Int.Tab. Vol. C (1992) p. 523,Tab. 6.3.3.3 for values of muR in the range 0-2.5, and Int.Tab. Vol.II (1959) p.302; Table 5.3.6 B for muR in the range 2.6-10.0. The interpolation procedure of (Dwiggins, 1975) is used with some modification
Geometry. All e.s.d.'s (except the e.s.d. in the dihedral angle between two l.s. planes) are estimated using the full covariance matrix. The cell e.s.d.'s are taken into account individually in the estimation of e.s.d.'s in distances, angles and torsion angles; correlations between e.s.d.'s in cell parameters are only used when they are defined by crystal symmetry. An approximate (isotropic) treatment of cell e.s.d.'s is used for estimating e.s.d.'s involving l.s. planes.
Refinement. Refinement of *F*^2^ against ALL reflections. The weighted *R*-factor *wR* and goodness of fit *S* are based on *F*^2^, conventional *R*-factors *R* are based on *F*, with *F* set to zero for negative *F*^2^. The threshold expression of *F*^2^ > σ(*F*^2^) is used only for calculating *R*-factors(gt) *etc*. and is not relevant to the choice of reflections for refinement. *R*-factors based on *F*^2^ are statistically about twice as large as those based on *F*, and *R*- factors based on ALL data will be even larger.

### Fractional atomic coordinates and isotropic or equivalent isotropic displacement parameters (Å^2^)

**Table:**

	*x*	*y*	*z*	*U*_iso_*/*U*_eq_	
Br1	0.16110 (5)	−0.03469 (5)	0.285194 (14)	0.07850 (19)	
S1	0.11800 (10)	0.09831 (9)	0.14388 (3)	0.0586 (2)	
O1	−0.4811 (3)	0.7429 (2)	0.08768 (9)	0.0684 (6)	
O2	−0.7294 (3)	0.5931 (3)	0.06893 (9)	0.0644 (6)	
H1	−0.769 (6)	0.671 (5)	0.0724 (17)	0.097*	
N1	−0.1430 (3)	0.0490 (2)	0.05969 (8)	0.0424 (5)	
N2	−0.1946 (3)	−0.0608 (2)	0.02100 (9)	0.0482 (5)	
N3	−0.0817 (3)	−0.1770 (3)	0.02891 (9)	0.0514 (5)	
N4	0.0451 (3)	−0.1480 (2)	0.07213 (8)	0.0484 (5)	
C1	−0.5529 (4)	0.6156 (3)	0.07638 (10)	0.0506 (6)	
C2	−0.4489 (3)	0.4647 (3)	0.07046 (10)	0.0462 (6)	
C3	−0.5338 (3)	0.3184 (3)	0.06132 (11)	0.0515 (6)	
H3	−0.6594	0.3124	0.0577	0.062*	
C4	−0.4328 (3)	0.1809 (3)	0.05749 (10)	0.0496 (6)	
H4	−0.4891	0.0819	0.0515	0.059*	
C5	−0.2460 (3)	0.1937 (3)	0.06285 (9)	0.0421 (5)	
C6	−0.1586 (3)	0.3385 (3)	0.07012 (11)	0.0495 (6)	
H6	−0.0334	0.3446	0.0722	0.059*	
C7	−0.2614 (4)	0.4750 (3)	0.07421 (11)	0.0507 (6)	
H7	−0.2048	0.5741	0.0795	0.061*	
C8	0.0051 (3)	−0.0061 (3)	0.09069 (10)	0.0442 (5)	
C9	0.2832 (4)	−0.0542 (4)	0.16947 (12)	0.0618 (8)	
H9A	0.2221	−0.1435	0.1838	0.074*	
H9B	0.3459	−0.0938	0.141	0.074*	
C10	0.4163 (4)	0.0194 (3)	0.21228 (11)	0.0568 (7)	
C11	0.3845 (4)	0.0329 (3)	0.26468 (11)	0.0567 (7)	
C12	0.5133 (4)	0.0977 (4)	0.30333 (13)	0.0699 (8)	
H12	0.4902	0.1031	0.3384	0.084*	
C13	0.6728 (5)	0.1533 (5)	0.29027 (15)	0.0862 (11)	
H13	0.7582	0.197	0.3164	0.103*	
C14	0.7079 (5)	0.1450 (6)	0.23877 (17)	0.0932 (12)	
H14	0.8159	0.1852	0.2297	0.112*	
C15	0.5817 (4)	0.0764 (5)	0.20012 (14)	0.0781 (10)	
H15	0.6081	0.0684	0.1654	0.094*	

### Atomic displacement parameters (Å^2^)

**Table:**

	*U*^11^	*U*^22^	*U*^33^	*U*^12^	*U*^13^	*U*^23^
Br1	0.0729 (3)	0.0918 (3)	0.0707 (3)	−0.00307 (17)	0.00932 (18)	0.00600 (16)
S1	0.0576 (4)	0.0567 (4)	0.0563 (4)	0.0182 (3)	−0.0120 (3)	−0.0109 (3)
O1	0.0681 (12)	0.0486 (11)	0.0884 (15)	0.0129 (10)	0.0105 (10)	−0.0114 (10)
O2	0.0531 (12)	0.0549 (12)	0.0823 (14)	0.0223 (9)	−0.0014 (10)	−0.0076 (10)
N1	0.0388 (10)	0.0404 (11)	0.0466 (11)	0.0060 (8)	0.0005 (8)	−0.0039 (8)
N2	0.0435 (11)	0.0446 (11)	0.0550 (12)	0.0048 (9)	0.0011 (9)	−0.0070 (9)
N3	0.0468 (11)	0.0477 (12)	0.0583 (13)	0.0048 (9)	0.0018 (9)	−0.0063 (10)
N4	0.0445 (11)	0.0464 (12)	0.0532 (12)	0.0101 (9)	0.0027 (9)	−0.0019 (9)
C1	0.0530 (15)	0.0498 (16)	0.0480 (14)	0.0154 (12)	0.0030 (11)	−0.0014 (11)
C2	0.0472 (14)	0.0459 (14)	0.0442 (13)	0.0136 (11)	0.0013 (10)	0.0011 (10)
C3	0.0391 (12)	0.0516 (14)	0.0623 (15)	0.0094 (11)	0.0007 (11)	−0.0028 (12)
C4	0.0414 (13)	0.0442 (13)	0.0610 (15)	0.0043 (10)	−0.0010 (11)	−0.0028 (11)
C5	0.0409 (12)	0.0413 (12)	0.0429 (12)	0.0101 (10)	0.0012 (9)	−0.0009 (9)
C6	0.0391 (12)	0.0458 (14)	0.0624 (15)	0.0063 (10)	0.0026 (11)	−0.0007 (11)
C7	0.0500 (14)	0.0413 (13)	0.0589 (15)	0.0015 (11)	0.0009 (12)	−0.0023 (11)
C8	0.0406 (12)	0.0457 (13)	0.0456 (13)	0.0090 (10)	0.0037 (10)	0.0001 (10)
C9	0.0615 (17)	0.0648 (18)	0.0545 (16)	0.0235 (14)	−0.0098 (13)	−0.0083 (13)
C10	0.0516 (16)	0.0634 (17)	0.0519 (15)	0.0204 (13)	−0.0066 (12)	−0.0011 (12)
C11	0.0564 (16)	0.0570 (16)	0.0538 (16)	0.0134 (12)	−0.0038 (12)	0.0022 (12)
C12	0.0649 (19)	0.080 (2)	0.0589 (17)	0.0110 (16)	−0.0149 (14)	−0.0054 (15)
C13	0.066 (2)	0.105 (3)	0.079 (2)	−0.0006 (19)	−0.0213 (18)	−0.0040 (19)
C14	0.0483 (18)	0.129 (3)	0.098 (3)	0.0027 (19)	−0.0050 (17)	0.013 (2)
C15	0.0581 (18)	0.113 (3)	0.0615 (19)	0.0226 (19)	0.0036 (15)	0.0099 (18)

### Geometric parameters (Å, º)

**Table:**

Br1—C11	1.896 (3)	C4—H4	0.93
S1—C8	1.728 (3)	C5—C6	1.374 (4)
S1—C9	1.830 (3)	C6—C7	1.386 (3)
O1—C1	1.208 (3)	C6—H6	0.93
O2—C1	1.318 (3)	C7—H7	0.93
O2—H1	0.72 (4)	C9—C10	1.502 (4)
N1—C8	1.349 (3)	C9—H9A	0.97
N1—N2	1.361 (3)	C9—H9B	0.97
N1—C5	1.440 (3)	C10—C11	1.386 (4)
N2—N3	1.283 (3)	C10—C15	1.394 (5)
N3—N4	1.371 (3)	C11—C12	1.388 (4)
N4—C8	1.324 (3)	C12—C13	1.358 (5)
C1—C2	1.498 (3)	C12—H12	0.93
C2—C3	1.381 (4)	C13—C14	1.368 (6)
C2—C7	1.392 (4)	C13—H13	0.93
C3—C4	1.384 (4)	C14—C15	1.389 (5)
C3—H3	0.93	C14—H14	0.93
C4—C5	1.386 (3)	C15—H15	0.93
			
C8—S1—C9	99.29 (12)	C2—C7—H7	119.9
C1—O2—H1	106 (4)	N4—C8—N1	107.7 (2)
C8—N1—N2	108.81 (18)	N4—C8—S1	128.29 (19)
C8—N1—C5	130.9 (2)	N1—C8—S1	124.04 (18)
N2—N1—C5	120.24 (19)	C10—C9—S1	108.74 (19)
N3—N2—N1	106.25 (19)	C10—C9—H9A	109.9
N2—N3—N4	111.12 (19)	S1—C9—H9A	109.9
C8—N4—N3	106.15 (19)	C10—C9—H9B	109.9
O1—C1—O2	124.2 (2)	S1—C9—H9B	109.9
O1—C1—C2	123.1 (2)	H9A—C9—H9B	108.3
O2—C1—C2	112.7 (2)	C11—C10—C15	117.0 (3)
C3—C2—C7	120.1 (2)	C11—C10—C9	123.1 (3)
C3—C2—C1	121.9 (2)	C15—C10—C9	119.9 (3)
C7—C2—C1	118.0 (2)	C10—C11—C12	121.3 (3)
C2—C3—C4	120.3 (2)	C10—C11—Br1	120.5 (2)
C2—C3—H3	119.9	C12—C11—Br1	118.2 (2)
C4—C3—H3	119.9	C13—C12—C11	120.5 (3)
C3—C4—C5	118.6 (2)	C13—C12—H12	119.8
C3—C4—H4	120.7	C11—C12—H12	119.8
C5—C4—H4	120.7	C12—C13—C14	120.0 (3)
C6—C5—C4	122.3 (2)	C12—C13—H13	120
C6—C5—N1	119.9 (2)	C14—C13—H13	120
C4—C5—N1	117.8 (2)	C13—C14—C15	119.9 (4)
C5—C6—C7	118.5 (2)	C13—C14—H14	120.1
C5—C6—H6	120.8	C15—C14—H14	120.1
C7—C6—H6	120.8	C14—C15—C10	121.4 (3)
C6—C7—C2	120.3 (2)	C14—C15—H15	119.3
C6—C7—H7	119.9	C10—C15—H15	119.3

### Hydrogen-bond geometry (Å, º)

Cg1 and Cg2 are the centroids of the N1–N4/C8 tetrazole ring and the C10–C15 benzene ring, respectively.

**Table:**

*D*—H···*A*	*D*—H	H···*A*	*D*···*A*	*D*—H···*A*
C9—H9*B*···O1^i^	0.97	2.41	3.351 (4)	163
O2—H1···N4^ii^	0.73 (4)	2.05 (4)	2.746 (3)	161 (5)
C1—O1···*Cg*1^iii^	1.21 (1)	3.62 (1)	4.534 (1)	133 (2)
C11—Br1···*Cg*2^iv^	1.90 (1)	3.58 (1)	4.895 (2)	124 (1)

Symmetry codes: (i) *x*+1, *y*−1, *z*; (ii) *x*−1, *y*+1, *z*; (iii) *x*, *y*+1, *z*; (iv) −*x*+1/2, *y*−1/2, −*z*+1/2.

## References

[bb1] Altomare, A., Cascarano, G., Giacovazzo, C., Guagliardi, A., Burla, M. C., Polidori, G. & Camalli, M. (1994). *J. Appl. Cryst.* **27**, 435.

[bb2] Dwiggins, C. W. (1975). *Acta Cryst.* A**31**, 146–148.

[bb3] Farrugia, L. J. (2012). *J. Appl. Cryst.* **45**, 849–854.

[bb4] Irwin, J. J., Sterling, T., Mysinger, M. M., Bolstad, E. S. & Coleman, R. G. (2012). *J. Chem. Inf. Model.* **52**, 1757–1768. 10.1021/ci3001277 PMC3402020 22587354

[bb5] Kees, K. L., Cheeseman, R. S., Prozialeck, D. H. & Steiner, K. E. (1989). *J. Med. Chem.* **32**, 11–13. 10.1021/jm00121a003 2642552

[bb6] Macrae, C. F., Bruno, I. J., Chisholm, J. A., Edgington, P. R., McCabe, P., Pidcock, E., Rodriguez-Monge, L., Taylor, R., van de Streek, J. & Wood, P. A. (2008). *J. Appl. Cryst.* **41**, 466–470.

[bb7] Mafud, A. C., Mascarenhas, Y. P. & Nascimento, A. S. (2013). *Acta Cryst.* E**69**, o759. 10.1107/S160053681300980X PMC3648285 23723905

[bb8] Nolte, R. T., Wisely, G. B., Westin, S., Cobb, J. E., Lambert, M. H., Kurokawa, R., Rosenfeldk, M. G., Willson, T. M., Glass, C. K. & Milburn, M. V. (1998). *Nature*, **395**, 137–143. 10.1038/25931 9744270

[bb9] Nonius (1999). *COLLECT* Nonius BV, Delft, The Netherlands.

[bb10] Otwinowski, Z. & Minor, W. (1997). *Methods in Enzymology*, Vol. 276, *Macromolecular Crystallography*, Part A, edited by C. W. Carter Jr & R. M. Sweet, pp. 307–326. New York: Academic Press.

[bb11] Sheldrick, G. M. (2008). *Acta Cryst.* A**64**, 112–122. 10.1107/S0108767307043930 18156677

